# Evaluation of the Colour Stability and Surface Roughness of Polymethylmethacrylate and Indirect Composites With and Without Ageing: An In-Vitro Study

**DOI:** 10.7759/cureus.68073

**Published:** 2024-08-28

**Authors:** Joshua Narde, Nabeel Ahmed, Varun Keskar, Kiran Kumar Pandurangan

**Affiliations:** 1 Department of Prosthodontics, Saveetha Dental College and Hospitals, Saveetha Institute of Medical and Technical Sciences, Saveetha University, Chennai, IND

**Keywords:** colour stability, dental materials, surface roughness, indirect composite resins, polymethylmethacrylate (pmma)

## Abstract

Background: Indirect restorations are a staple restorative option in dentistry due to their versatility, exceptional aesthetics, and desirable strength and longevity. Metal ceramics and all ceramics are the material of choice for permanent restorations but come with certain disadvantages, such as chipping, fracture, and wear of the antagonist tooth or restoration. Polymethylmethacrylate (PMMA) and indirect composite resins are commonly used materials due to their favourable properties for temporary restorations, but lately, they have been chosen as the restorative material of choice for implant-supported full-mouth rehabilitations. This study aimed to evaluate and compare the colour stability and surface roughness of PMMA and indirect composite resins under both ageing and non-ageing conditions. This would greatly help a clinician in choosing materials depending on the clinical scenarios.

Aim: This study aims to evaluate the colour stability and surface roughness of PMMA and indirect composite resins with and without ageing.

Methods: Specimens of PMMA and indirect composite resins were fabricated and subjected to an ageing process involving thermocycling in the TW-C4.4 (Tae-Won Tech, Incheon, Korea) and immersion in a staining solution. Colour stability was assessed using a spectrophotometer (SpectraMagic NX, RM2002QC, Konica Minolta Corp., Ramsey, Japan), and surface roughness was measured using a stylus profilometer (Mituyoto, Mituyoto Corporation, Kawasaki, Japan). Statistical analysis was performed using IBM SPSS Statistics for Windows, Version 24 (Released 2016; IBM Corp., Armonk, New York) to determine significant differences between materials and ageing effects.

Results: PMMA exhibited significantly higher colour changes and increased surface roughness post-ageing compared to indirect composite resins. The findings underscored PMMA's susceptibility to discolouration and surface degradation under thermal stress conditions.

Conclusion: PMMA demonstrates inferior colour stability and increased surface roughness following thermocycling compared to indirect composite materials, suggesting careful consideration in material selection for provisional restorations. Further research should explore additional ageing processes and materials to enhance understanding and improve clinical outcomes.

## Introduction

The current trends in restorative dentistry involve using highly durable materials that are resistant to dimensional changes and display the least deleterious effects on the antagonist teeth or restorations [1,2]. A variety of restorative materials can be used for this process. Materials such as metals and ceramics are the most commonly used. Indirect composite resins, polymethylmethacrylate (PMMA), polyetheretherketone (PEEK), and graphene are newer materials that are predominantly used as temporary restorations but are now being considered as materials for final prostheses due to their versatility. Provisional restorations play a crucial role in dental treatments, serving as temporary solutions that protect prepared teeth, maintain aesthetics, and ensure proper function until definitive restorations are placed. The materials have performed exceedingly well, which makes us wonder about their efficacy as permanent restorations. Among the various materials available, PMMA and indirect composite resins are widely used due to their favourable properties [3-5]. However, the long-term success of these materials depends significantly on their ability to maintain colour stability and surface integrity over time, particularly when subjected to the dynamic and often harsh conditions of the oral environment [6,7].

Colour stability is a fundamental aesthetic requirement for restorations. Colour changes can lead to patient dissatisfaction and may necessitate premature restoration replacement, thereby increasing treatment costs and time [8,9]. PMMA has been extensively used in dental practice due to its ease of manipulation, cost-effectiveness, and satisfactory aesthetic properties. However, it is susceptible to discolouration, especially when exposed to common staining agents found in foods and beverages [10]. Indirect composite resins, on the other hand, offer enhanced mechanical properties and improved aesthetics. Yet, they are not immune to colour changes, particularly under the influence of various ageing processes, due to the presence of micro-porosities, which are present in their structural configuration [11].

Surface roughness is another critical factor influencing the clinical performance of restorations. A smooth surface not only contributes to the aesthetic appearance but also minimizes plaque accumulation, thereby reducing the risk of periodontal disease and secondary caries [12,13]. The oral environment, characterized by fluctuating pH levels, varying temperatures, and mechanical wear from mastication and brushing, can significantly affect the surface texture of restorative materials [14]. PMMA restorations, despite their popularity, tend to exhibit increased surface roughness over time, which can compromise their aesthetic and functional performance by harbouring more microorganisms [15]. Indirect composite resins, although generally more resistant to surface wear, can also experience changes in surface roughness due to similar environmental factors [16].

The process of artificial ageing is commonly employed in research to simulate the long-term effects of oral conditions on dental materials within a shortened timeframe. This involves exposing the materials to accelerated conditions, such as thermal cycling, UV light exposure, and immersion in staining solutions, to mimic the effects of prolonged use [17,18]. Understanding how PMMA and indirect composite resins respond to such ageing processes is essential for predicting their long-term performance and guiding clinicians in selecting the right material. With a variety of prostheses available for implant-supported frameworks and restorations, these materials are sure to make a big difference when it comes to choosing restorative materials.

Despite the widespread use of PMMA and indirect composites, there is a paucity of comprehensive studies directly comparing their colour stability and surface roughness under both ageing and non-ageing conditions. The main aim of this study is to evaluate the colour stability and surface roughness with and without ageing. The null hypothesis is that there is no difference in the colour stability and surface roughness of PMMA and indirect composite resin before and after ageing. By systematically comparing these properties, the study seeks to provide valuable insights into the durability and aesthetic longevity of these materials, ultimately aiding clinicians in making informed decisions regarding the choice of restorative materials when delivering both temporary and permanent restorations.

## Materials and methods

Sample size determination

This study was approved by the Institutional Review Board and was conducted at the Department of Prosthodontics at a private dental college in Chennai, India. The sample size was ascertained by consulting data from an earlier publication using G*Power 3.1.9.3 for Mac OS X® (Heinrich-Heine-Universität Düsseldorf, Düsseldorf, Germany). A power of 0.95 (1−β error probability), an effect size (dz=1.5004), and a significance of 0.05 (α) were confirmed for the intervention. A final sample size of 72 was determined for the study [19].

Specimen preparation

**Figure 1:**
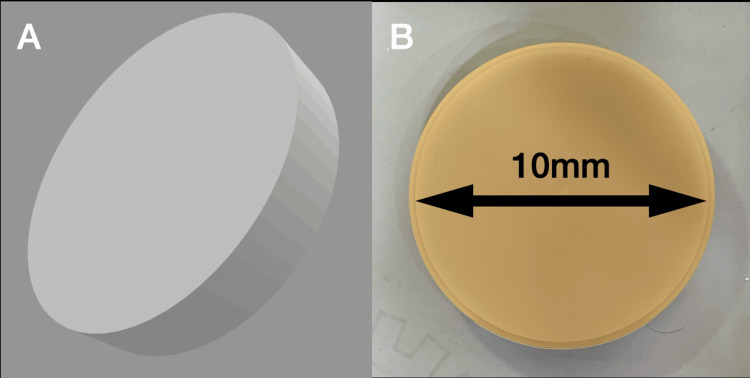
Polymethylmethacrylate (PMMA) disc and designed STL file. Figure 1A is the STL file created for the PMMA disc. Figure 1B is the milled PMMA disc with a 10 mm diameter.

A total of 72 disc-shaped specimens (10×2 mm) of both materials were prepared. Group 1 comprised 36 samples of PMMA (Figure 1), and Group 2 consisted of 36 samples of indirect composite resin. The objects were made to resemble discs. Using computer-aided design, an STL file of the abovementioned dimensions was readied. This was then nested in a PMMA disc (Upcera, Shenzhen Upcera Dental Technology Co., Ltd., Guangdong, China), also referred to as a blank or puck. Once completed, it was subjected to the milling procedure in the IMES iCore milling unit (CORiTEC 350i, Eiterfeld, Germany). The specimens were polished using a series of silicon carbide burs to achieve the final finish after milling. First, a tungsten carbide bur was used to remove any excess resin attached to the specimens. Finishing was done with silicon polishing burs of various grits (600-grit, 800-grit, 1,000-grit, and 1,200-grit). It was ultimately polished with a cloth wheel and pumice. Only one surface was polished to simulate intra-oral conditions.

**Figure 2:**
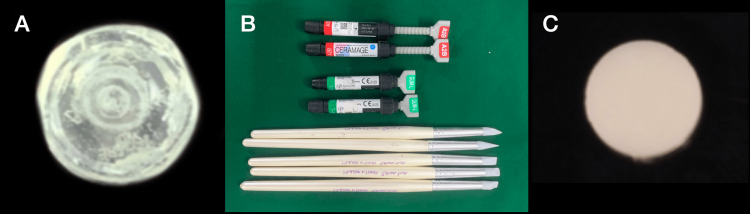
Silicone mould fabrication followed by indirect composite disc preparation after polishing. Figure 2A shows the fabricated silicone mould. Figure 2B shows the indirect composite resin. Figure 2C shows the final cured composite disc.

The indirect composite( Shofu Ceramage, SHOFU Dental GmbH, Ratingen, Germany) was layered into a silicone mould with a diameter of the disc size and light-cured until a complete set was achieved (Figure 2). The light curing was done in the Shofu Solidite V (SHOFU Dental GmbH) curing chamber. This curing chamber comprises four powerful halogen lamps of 150 W each. The wavelength light spectrum is 400-550 nm for each bulb, which assures proper curing within the chamber. The distance can be adjusted depending on the selected level of the turntable. The curing time varies from one to five minutes, depending on the chosen setting. A three-minute curing cycle was chosen. The benefit of this system is that it follows gentle curing, which is ideal for this material in the chamber. After curing, the correct polishing sequence was followed per the manufacturer's instructions, which included silicone burs and a buff to achieve a polished surface. This ensured consistency among all the specimens, and uniformity was maintained. The polishing was done only on one surface.

Ageing procedure

**Figure 3:**
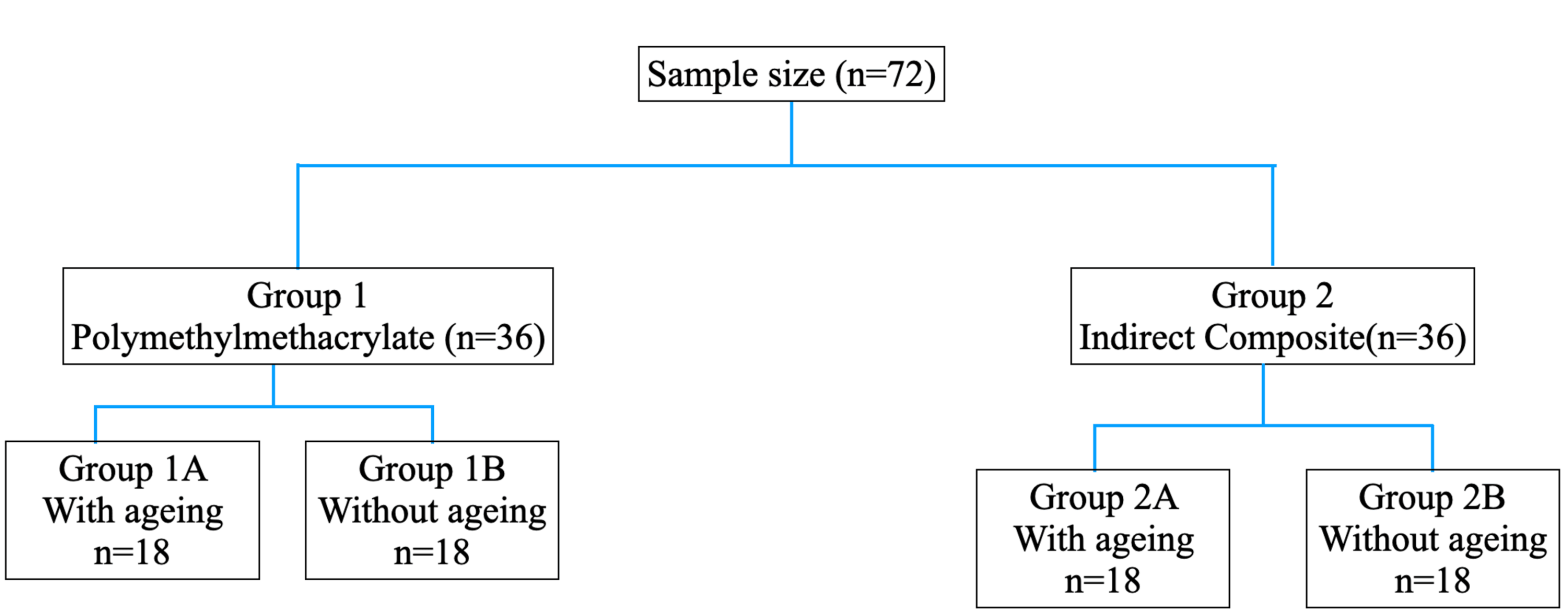
Descriptive flowchart of the steps followed.

The specimens were further divided into two groups: Group 1A and 1B and Group 2A and 2B. Group 1A and 2A underwent the ageing procedures, whereas Group 1B and 2B were not subjected to ageing. Figure 3 presents a tabular flowchart of the steps that were followed. Half of the specimens from each material group were subjected to an ageing process to simulate long-term use in the oral environment. The ageing process involved thermocycling and immersion in a staining solution. The specimens underwent 5000 thermocycles in a dual-axis chewing simulator TW-C4.4 (Tae-Won Tech, Incheon, Korea) between 5 °C and 55 °C with a dwell time of one minute at each temperature and a transfer time of five seconds [19]. This process aimed to simulate the thermal stresses experienced by restorative materials in the oral cavity. Following thermocycling, the aged specimens were immersed in a coffee solution for seven days to evaluate their colour stability, which would be equivalent to one year. As directed by the manufacturer, one tablespoon of coffee was added to 177 mL of water in a filter coffee maker to create the coffee solution [20]. Every 12 hours, the coffee solution was refreshed to maintain the same concentration throughout.

Surface roughness and colour stability evaluation

**Figure 4:**
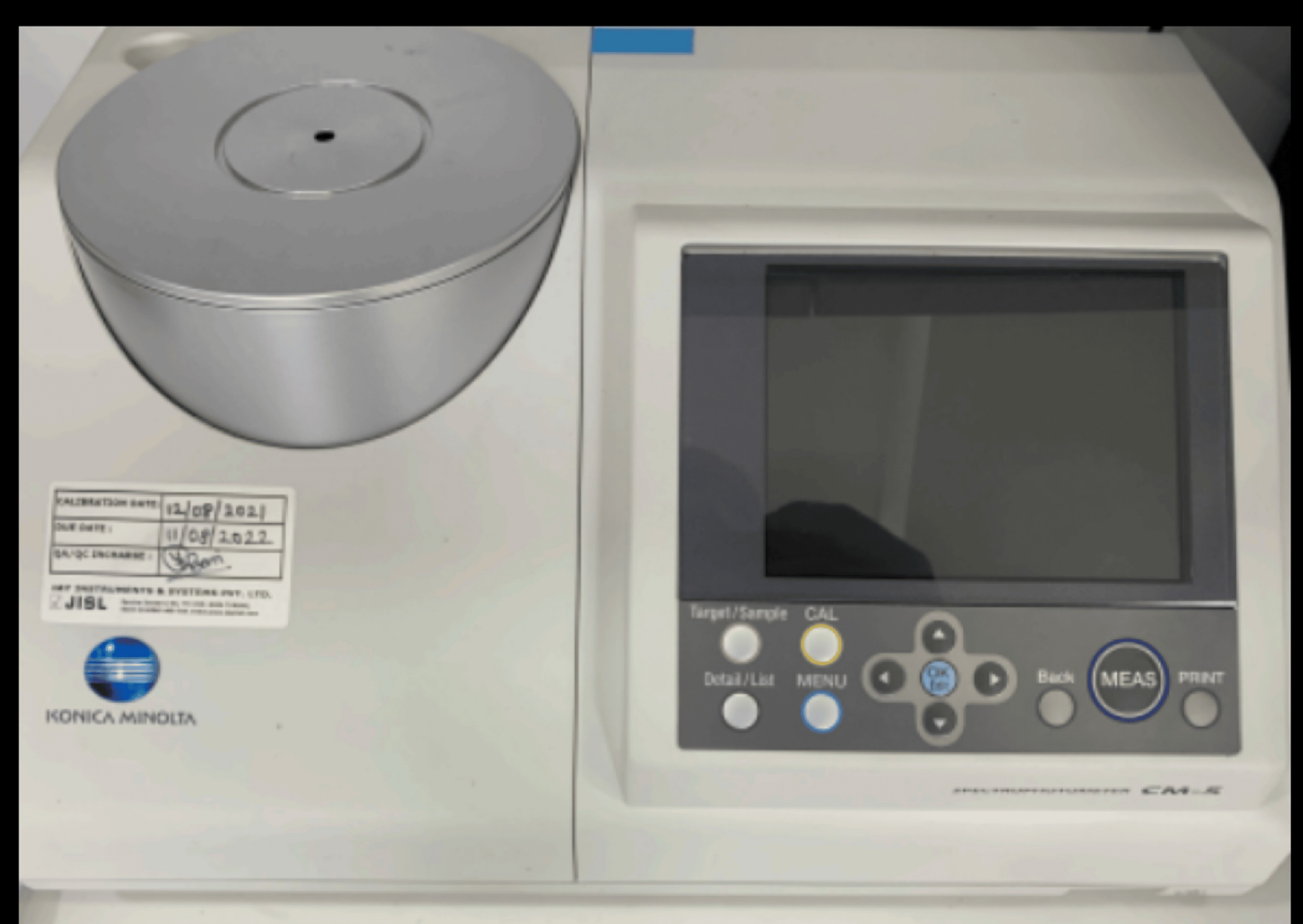
Spectrophotometer (Konica Minolta).

**Figure 5:**
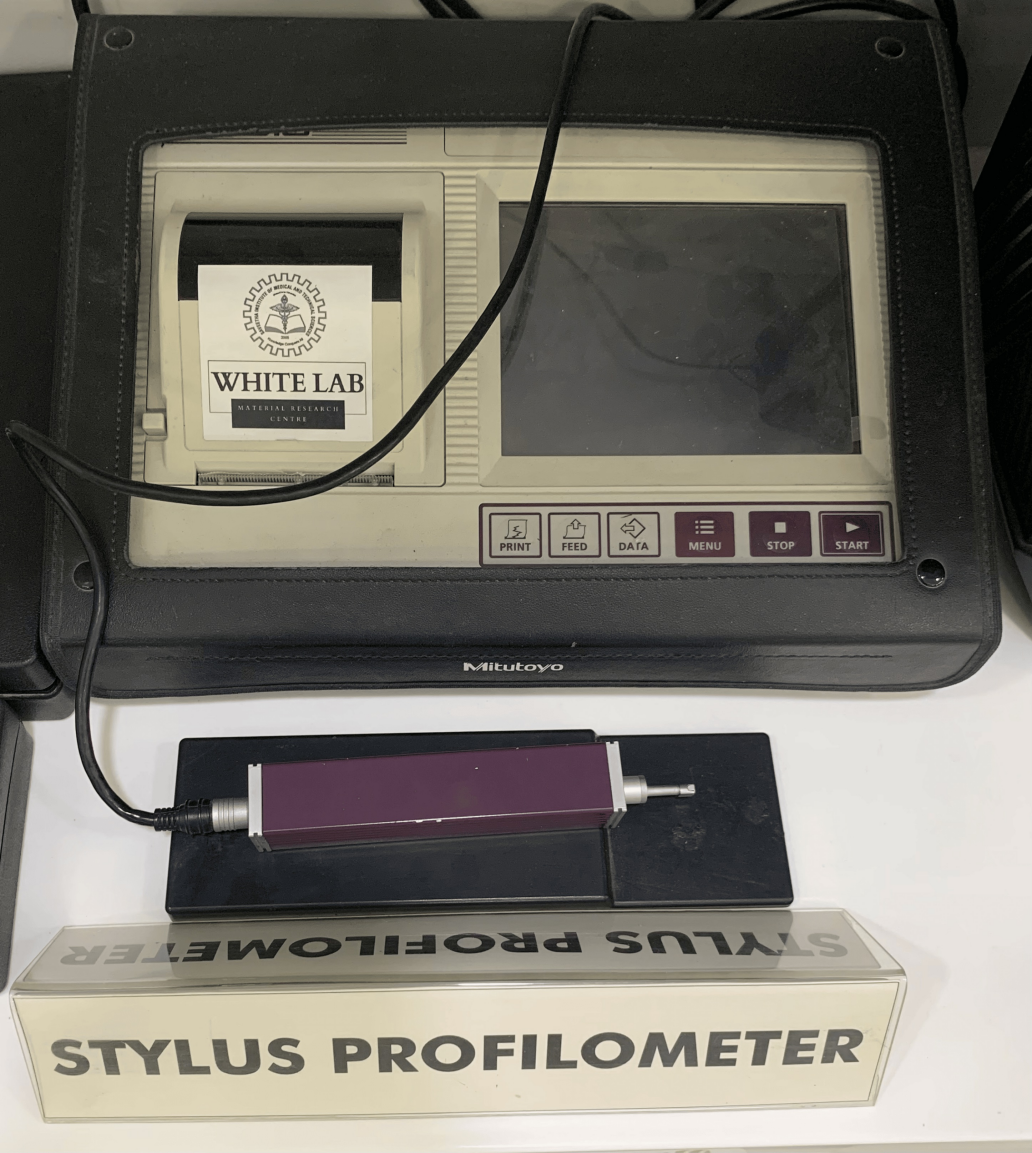
Stylus profilometer used to test the surface roughness.

The colour stability of each specimen was measured using a spectrophotometer (SpectraMagic NX, RM2002QC, Konica Minolta Corp., Ramsey, Japan) (Figure 4). The measurements were recorded in the machine name colour space, and the colour difference was calculated before and after ageing. The surface roughness and colour stability of all specimens were evaluated before and after the ageing process. The surface roughness of each specimen was measured using a stylus profilometer by Mitutoyo (Mitutoyo Corporation, Japan) (Figure 5). Before taking any readings, the profilometer was calibrated on the "Precision Reference Specimen" provided by the company. This removes any error that could be present before taking the new readings. Three measurements were taken at different points on each specimen, and the average value was recorded.

Statistical analysis

The data were analyzed using IBM SPSS Statistics for Windows, Version 24 (Released 2016; IBM Corp., Armonk, New York). The mean and standard deviation for surface roughness and colour stability were calculated. Normality was tested using the Kolmogorov-Smirnov test. The paired sample t-test was carried out to evaluate the colour stability and surface roughness before and after the ageing process. An independent t-test was used to compare the PMMA and indirect composite resin groups.

## Results

Colour stability

**Figure 6:**
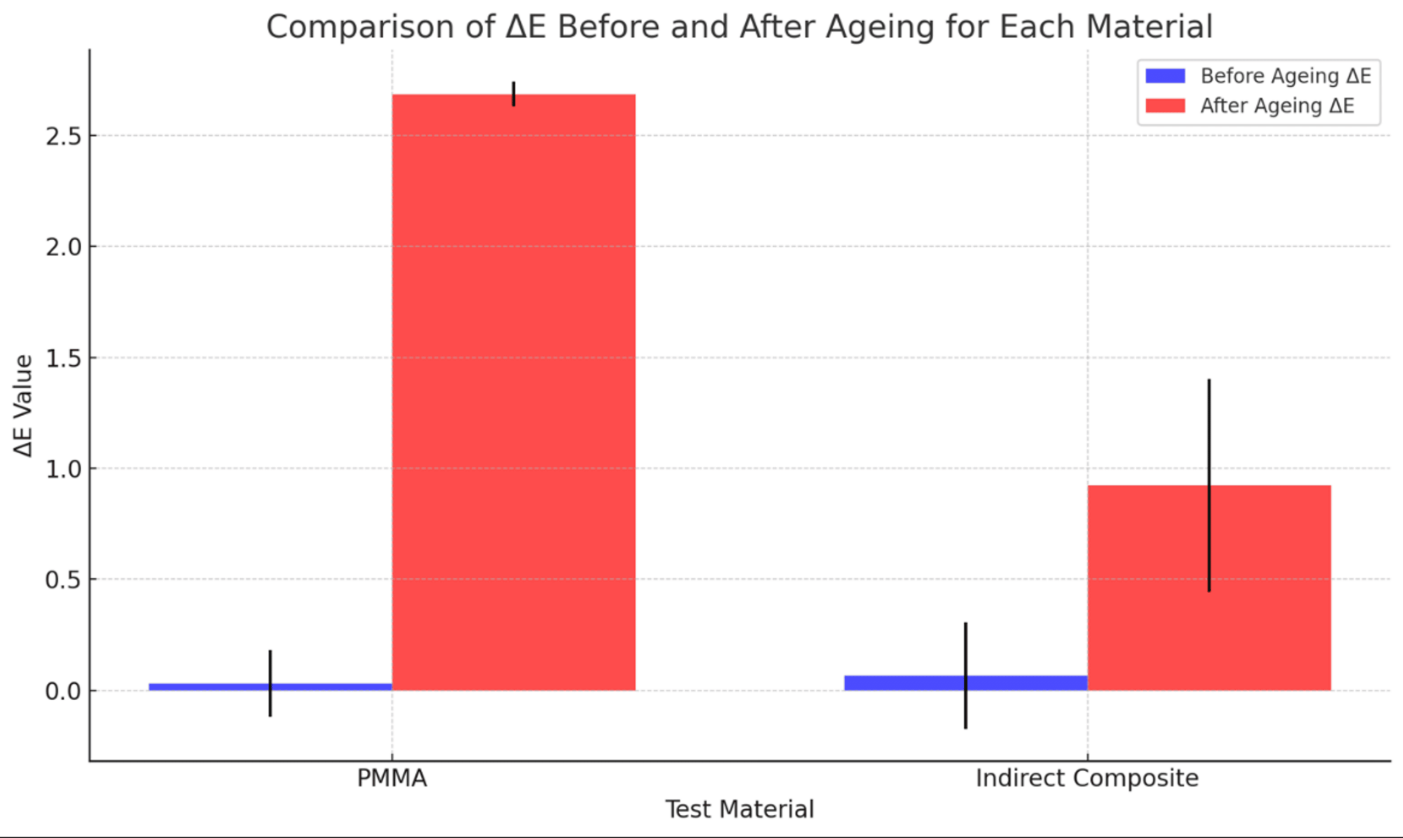
Graphical representation of the materials before and after ageing.

The colour stability of the materials was evaluated by measuring the colour changes (ΔE) after the ageing process. The mean ΔE values indicated that both PMMA and indirect composite materials experienced colour changes, but the extent varied between the materials and ageing conditions. Table 1 contains the ΔE values before and after the process for PMMA and indirect composites. The ΔE value for PMMA after ageing was 2.687 ± 0.056 (p = 0.002), which shows the difference from the values obtained before the ageing procedure. The ΔE values under 3.3 are considered acceptable for restorations, after which changes are seen in the restoration performance. After 5000 cycles of ageing, which is equivalent to one year of intraoral time, there was a significant change, which is seen in the case of PMMA, although this change is considered within acceptable limits. The ΔE values for indirect composite resin after ageing are 0.923 ± 0.481 (p = 0.032). Figure 6 presents a graphical representation of the ΔE values before and after ageing. PMMA's very low p-value (0.002) suggests a highly significant change in ΔE after ageing. The p-value (0.032) for indirect composite also indicates a change, although less intense than for PMMA regarding colour stability.

**Table 1:** Colour stability measured using the spectrophotometer; ΔE values compared.

Test Material	Before Ageing ΔE	After Ageing ΔE	p-value
PMMA	0.03 ± 0.15	2.687 ± 0.056	0.002
Indirect composite	0.067 ± 0.24	0.923 ± 0.481	0.032

Surface roughness

**Figure 7:**
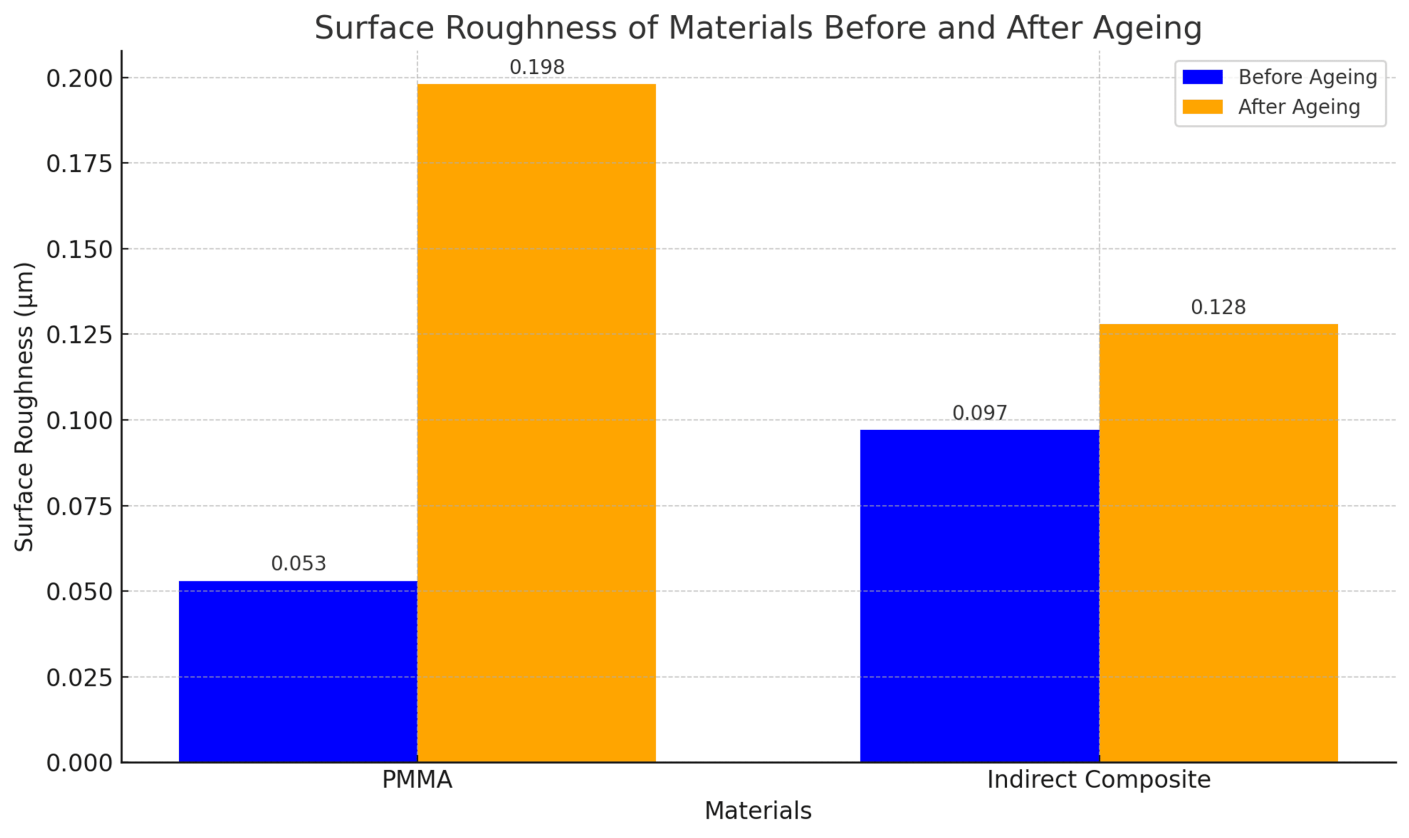
Graphical representation of surface roughness of PMMA and indirect composite before and after ageing.

The roughness values (Ra) before and after the ageing process were compared to understand the impact of ageing on the surface texture of the materials (Table 2). The PMMA samples exhibited a higher increase in Ra values post-ageing compared to the indirect composite, suggesting that PMMA is more susceptible to surface deterioration over time. The surface roughness of the PMMA was 0.198 ± 0.41 µm (p = 0.16), whereas that of the indirect composite was 0.128 ± 0.36 µm (p = 0.28). The change in the surface roughness is found to be insignificant in the case of both materials after the ageing process. Although the PMMA shows more change as compared to the indirect composite, the materials do not show significant deterioration after 5000 cycles of thermocycling. Figure 7 depicts the graphical representation of the data.

**Table 2:** Surface roughness values.

Test Material	Before Ageing (µm)	After Ageing (µm)	Mean Difference (µm)	p-value
PMMA	0.053 ± 0.022	0.198 ± 0.41	0.0019 ± 0.007	0.16
Indirect composite	0.097 ± 0.018	0.128 ± 0.36	0.023 ±0.004	0.28

## Discussion

The findings from the study reveal that PMMA exhibits increased surface roughness and reduced colour stability following thermocycling compared to indirect composite materials. These results are critical in understanding the long-term performance of materials used for restorations in clinical practice.

The increased surface roughness observed in PMMA after thermocycling can be attributed to its inherent material properties and the impact of thermal stress [19]. PMMA, being a thermoplastic resin, is susceptible to changes in temperature, which can cause alterations in its surface morphology. The repeated exposure to extreme temperatures during thermocycling is more likely to accentuate these changes, resulting in a rougher surface [21,22]. These findings align with previous studies, such as those by Ayaz et al. (2015) and Atalay et al. (2021), which reported similar trends in surface roughness for PMMA under thermal stress conditions. The clinical implications of increased surface roughness are significant, as it can lead to higher plaque accumulation, increased wear, and compromised aesthetics, ultimately affecting the longevity and performance of the restoration [21,23].

The results of this study are consistent with those reported by Çakmak et al. (2023), who investigated the surface roughness and stainability of nanographene-reinforced PMMA compared to prepolymerized PMMA and reinforced composite resin after coffee thermocycling. Çakmak et al. found that while PMMA had higher surface roughness before polishing, the differences after polishing and coffee thermocycling were not significant. However, their study also noted that PMMA exhibited higher Ra values compared to reinforced composite resin, aligning with our findings of increased roughness in PMMA. Furthermore, Çakmak et al. concluded that coffee thermocycling increased the colour change values of all materials tested, corroborating our results that thermocycling negatively affects PMMA's colour stability [24]. Similarly, Ayaz et al. (2015) evaluated the effects of thermal cycling on the surface roughness, hardness, and flexural strength of denture resins, including PMMA. Their findings revealed significant differences in surface roughness and mechanical properties before and after thermal cycling. Ayaz et al. reported that thermal cycling did not change the surface roughness of PMMA significantly, which contrasts with our findings of increased roughness. This discrepancy may be due to differences in the thermocycling protocols or the specific types of PMMA tested [22]. However, the general trend of PMMA showing greater susceptibility to surface changes compared to other materials remains consistent across studies.

In contrast, the indirect composite materials demonstrated better resistance to surface roughness changes under the same thermocycling conditions [25]. Indirect composites are typically composed of a combination of resin matrix and inorganic fillers and are designed to withstand mechanical and thermal stresses more effectively than PMMA. The inclusion of inorganic fillers in the composite resin enhances its mechanical properties, providing greater stability and resistance to surface degradation [26,27]. The reduced colour stability of PMMA after thermocycling further underscores its limitations for long-term provisional applications. Colour stability is a critical aesthetic parameter, as discolouration can lead to patient dissatisfaction and necessitate premature restoration replacement. The findings suggest that PMMA is more prone to discolouration, likely due to its porous nature and higher water absorption capacity, which facilitates the penetration of staining agents. This is corroborated by previous studies, including those by Banu et al. (2020) and Gujjari et al. (2013), which have highlighted the susceptibility of PMMA to staining and discolouration over time [28,29].

The clinical relevance of these findings is significant as they provide valuable insights for material selection for restorations, be it provisional or final restoration. While PMMA remains a popular choice due to its ease of use and cost-effectiveness, its susceptibility to increased surface roughness and reduced colour stability under thermal stress conditions necessitates careful consideration. With their superior performance in these aspects, indirect composite materials present a more durable and aesthetically reliable alternative for restorations [3,30,31]. Both these materials are increasingly being used in implant-supported full-mouth reconstructions. Hence, the null hypothesis has been rejected, stating that there is no difference between the surface roughness and colour stability of PMMA and indirect composite resin.

The study's limitations mainly include the fact that it is an in-vitro study following ideal conditions. Clinical trials need to be done to assess the performance and durability of these materials. Future research should focus on exploring the performance of a broader range of materials under various ageing processes to provide a more comprehensive understanding of their long-term clinical behaviour. Additionally, investigating the underlying mechanisms of surface roughness and colour changes in these materials can further inform the development of more robust and durable provisional restoration materials.

## Conclusions

From the limitations of this study, it can be concluded that PMMA demonstrates increased surface roughness and reduced colour stability following thermocycling compared to indirect composite resin. These findings indicate that while PMMA remains a popular choice due to its ease of use and cost-effectiveness, its long-term aesthetic and surface properties are more adversely affected by ageing processes. The indirect composite resin, on the other hand, displays better colour stability and surface roughness properties.
